# Cortexa: a comprehensive resource for studying gene expression and alternative splicing in the murine brain

**DOI:** 10.1186/s12859-024-05919-y

**Published:** 2024-09-05

**Authors:** Stephan Weißbach, Jonas Milkovits, Stefan Pastore, Martin Heine, Susanne Gerber, Hristo Todorov

**Affiliations:** 1 Institute of Developmental Biology and Neurobiology (iDN), Johannes Gutenberg University Mainz, 55128 Mainz, Germany; 2 Institute of Human Genetics, University Medical Center, Johannes Gutenberg University Mainz, 55131 Mainz, Germany; 3 Institute of Pharmaceutical and Biomedical Sciences, Johannes Gutenberg University Mainz, 55128 Mainz, Germany

**Keywords:** Alternative splicing, Gene expression, Cerebral cortex, Hippocampus, Embryonic brain development

## Abstract

**Background:**

Gene expression and alternative splicing are strictly regulated processes that shape brain development and determine the cellular identity of differentiated neural cell populations. Despite the availability of multiple valuable datasets, many functional implications, especially those related to alternative splicing, remain poorly understood. Moreover, neuroscientists working primarily experimentally often lack the bioinformatics expertise required to process alternative splicing data and produce meaningful and interpretable results. Notably, re-analyzing publicly available datasets and integrating them with in-house data can provide substantial novel insights. However, such analyses necessitate developing harmonized data handling and processing pipelines which in turn require considerable computational resources and in-depth bioinformatics expertise.

**Results:**

Here, we present Cortexa—a comprehensive web portal that incorporates RNA-sequencing datasets from the mouse cerebral cortex (longitudinal or cell-specific) and the hippocampus. Cortexa facilitates understandable visualization of the expression and alternative splicing patterns of individual genes. Our platform provides SplicePCA—a tool that allows users to integrate their alternative splicing dataset and compare it to cell-specific or developmental neocortical splicing patterns. All standardized gene expression and alternative splicing datasets can be downloaded for further in-depth downstream analysis without the need for extensive preprocessing.

**Conclusions:**

Cortexa provides a robust and readily available resource for unraveling the complexity of gene expression and alternative splicing regulatory processes in the mouse brain. The data portal is available at https://cortexa-rna.com/

## Background

Transcriptional regulation plays a crucial role in the developing and adult mammalian brain [1–3]. Major changes during development can be observed particularly in gene expression [4, 5] and alternative splicing [6–9] patterns.

Gene expression is the best-understood, and most extensively studied among these processes. It has been a major focus in the study of brain development [10], cell identity [11, 12], and disease-related alterations [13–15]. However, a common issue with gene expression analysis when comparing results from heterogenous sources is the occurrence of batch effects that are unrelated to biological factors, due to differences in sequencing technology, experimental handling, and bioinformatic processing [16–20].

Alternative splicing (AS) of precursor mRNA is a fundamental process that enables the generation of various transcripts and subsequent proteins from the same gene. Therefore, it significantly increases the available transcriptomic variability [21]. AS is particularly important in the central nervous system, playing a vital role during cortical development [6, 21] and in the determination and maintenance of neuronal cell identity [22]. Moreover, AS is an important regulator of gene expression since it can initiate the degradation of mRNA by introducing premature stop codons which leads to nonsense-mediated decay [23].

Although several high-quality datasets focusing on AS in the murine brain are freely available, interpreting the different types of splicing events for individual genes remains challenging. Moreover, harnessing the potential of multiple datasets requires a harmonized processing strategy to ensure the comparability of results. A limitation of existing data portals, namely, the single cell atlas of the Allen Brain Institute [24], Neuron Subtype Transcriptome [25], or Brain RNA-Seq [26], is that they are focused on gene expression and do not offer the analysis of custom data in the context of cell-specific or developmental changes in alternative splicing.

**Fig. 1:**
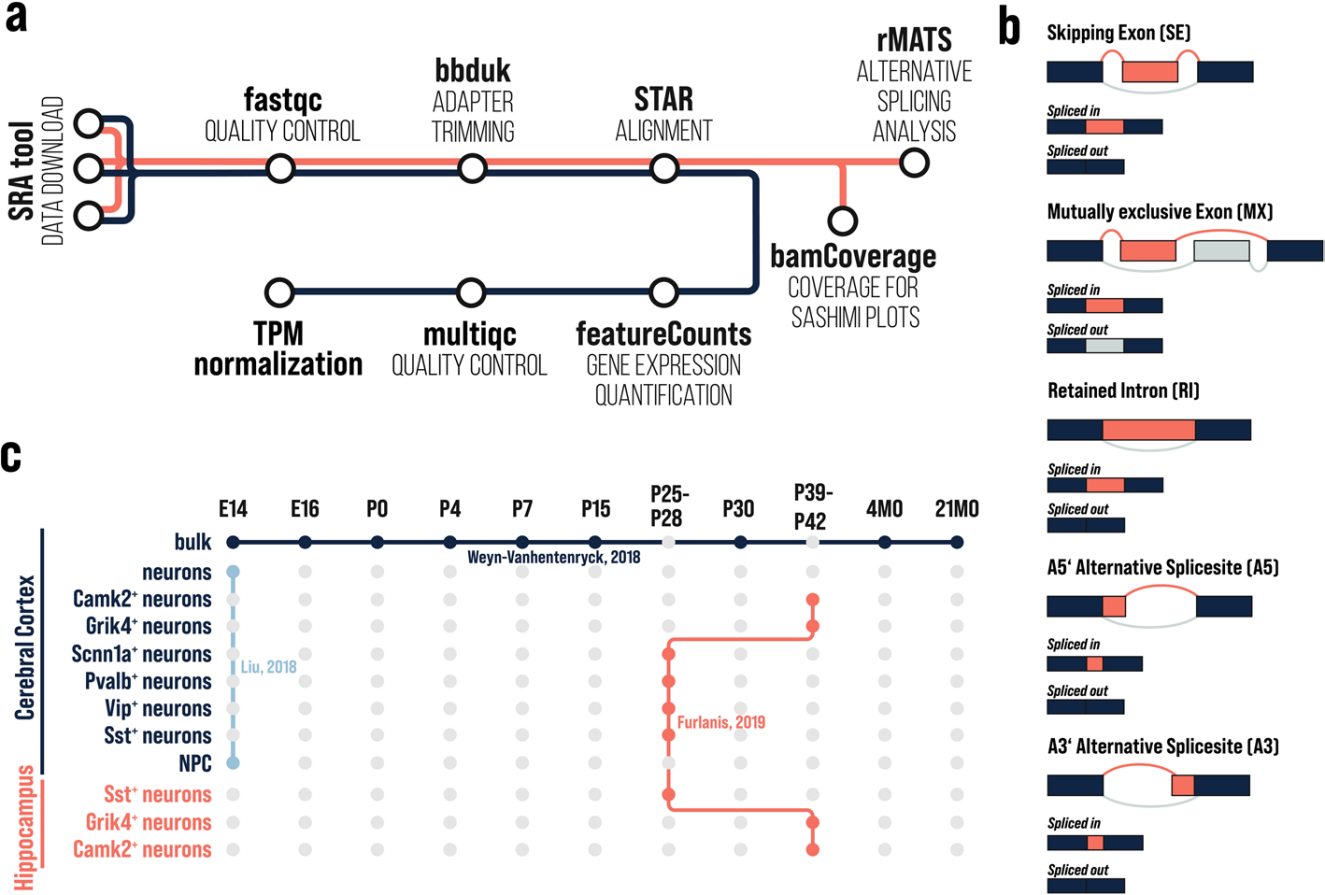
**a** Standardized RNA-seq pipeline used for gene expression (blue) and alternative splicing (orange). **b** Visualization of possible alternative splicing events and the splicing outcome referred to as spliced-in and spliced-out. **c** Publicly available datasets integrated into Cortexa: SRP055008 [6], GSE133291 [22], and GSE96950 [9]

Here, we introduce Cortexa—a novel data portal for accessing a variety of high-quality neocortical and hippocampal transcriptomic datasets, analyzed for gene expression and alternative splicing. Batch effects between different studies have been minimized using a standardized analysis pipeline ( Figure1a). We offer easily interpretable summaries and visualization of results that allow a broad range of scientists to explore the expression and AS patterns of individual genes. Additionally, we developed SplicePCA—a tool that performs a principal component analysis of splicing events for a selected gene set and enables the investigation of splicing patterns related to developmental changes or variations across cell types [6, 27–29]. All standardized datasets included in Cortexa are publicly available for download, allowing users to integrate them into their research easily.

## Construction and content

### Datasets

We analyzed publicly available in vivo paired-end RNA sequencing data of the mouse cerebral cortex and hippocampus as well as in vivo data from neural progenitor cells (NPCs) and neurons (Fig. 1c) with a minimum read length of 100 bp. We downloaded the sequencing data from NCBI SRA or GEO respectively. Specifically, we used SRP055008 [6], GSE133291 [22], and GSE96950 [9]. Further datasets can easily be integrated, refer to https://cortexa-rna.com/datasets.

### RNA-seq analysis

We used a standardized RNA-seq pipeline (Fig. 1a) to analyze the transcriptomic data for gene expression and alternative splicing. In brief, we trimmed the reads for adapter sequences with BBDuk (version 39.01) [30]. The trimmed reads were aligned to the reference genome mm39 (released 19.10.2022) downloaded from Gencode using STAR (version 2.7.10b) [31] and indexed using samtools (version 1.18) [32]. FeatureCounts, provided by SubRead (version 2.0.6) [33], was used to quantify the expression of each respective gene. All gene expression counts were normalized to transcripts per million (TPM).

We utilized rMATS turbo (version 4.1.2) [34] with default settings to detect AS events. We analyzed the data for five alternative splicing events: cassette exon (skipping exon), mutually exclusive exons, intron retention, alternative A5′ splice site, and alternative A3′ splice site (Fig. 1b), as defined in rMATS [34]. The coverage for sashimi plots was analyzed in 10 bp steps with bamCoverage (version 3.5.2) from deepTools2 [35] converted to wig with Encode bigWigToWig.

### Webapp

The web application was built with Next.js frontend framework, utilizing SQLite database for backend data storage. TypeScript enhances code maintainability and type safety, while Tailwind CSS streamlined styling. Prisma serves as the ORM tool for efficient database management. The application follows the REST principles for communication between frontend and backend components, optimizing interoperability and scalability. The website is hosted on servers of the Johannes Gutenberg University, Mainz, Germany. A detailed tutorial on how to interpret alternative splicing events presented in Cortexa is provided at https://cortexa-rna.com/tutorial.

### Visualization of genes

Gene expression was normalized to transcripts per million (TPM) and represented as a barplot for each dataset. Alternative splicing events (Fig. 1b) are visualized as sashimi plots.

### SplicePCA

**Fig. 2:**
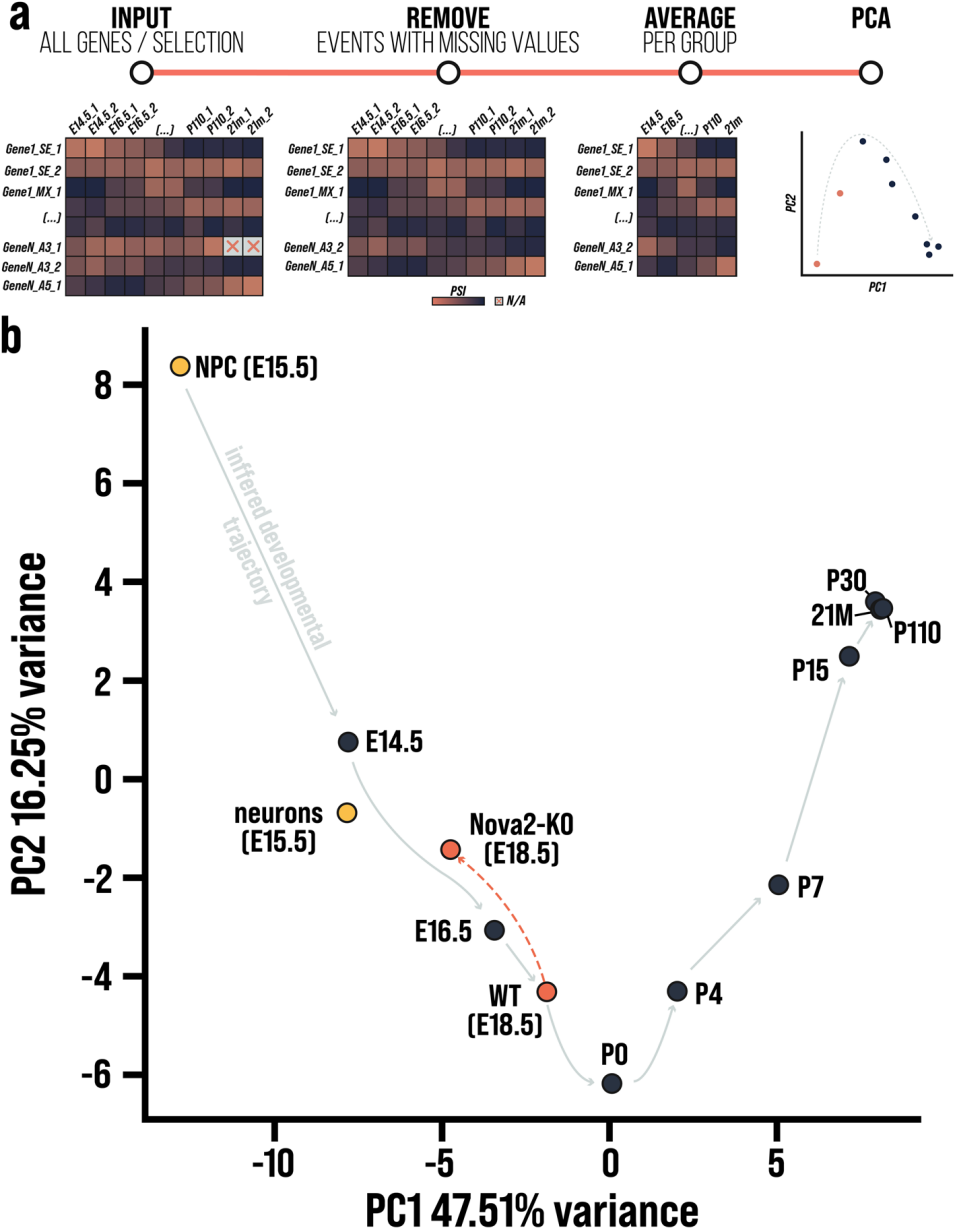
**a** Schematic representation of SplicePCA. SplicePCA takes percentage spliced-in (PSI) values for all genes or a selected subset of genes as input. Alternative spicing events with missing values are removed from the dataset. The remaining PSI values are averaged over individual replicates per experimental group. Finally, PCA is performed on the averaged data, resulting in a representation of splicing patterns across groups in two-dimensional space. **b** *Cassette exon data of cortical samples of WT (E18.5) and Nova2-KO (E18.5), analyzed with SplicePCA and compared to splicing changes across normal development of the mouse cortex. PCA of alternative splicing data across cortical development forms a characteristic bell-shaped trajectory (indicated by gray arrows) with P0 as its inflection point. The conditional knock-out of Nova2 resulted in a substantial deviation from the inferred normal splicing trajectory. Nova2-KO samples (E18.5) were associated with a less mature splicing pattern than E16.5 wild-type samples*

SplicePCA performs principal component analysis (PCA) on averaged percentage spliced-in (PSI) values (Fig. 2a). Initially, the user can either select a subset of genes or perform SplicePCA on all genes. All events that have missing values are removed from the subsequent analysis. Next, PSI values are averaged within their respective group (e.g. E14.5 from the developmental data set [6]). These averaged PSI values are the input for the PCA, and the resulting values are plotted and available for download. Moreover, SplicePCA allows users to integrate their in-house analyzed output files (rMATS). We recommend processing the files as described in Sect. 3.2 and in the tutorial available at https://github.com/s-weissbach/cortexa_SplicePCA_example/.

### Example usage of SplicePCA

To demonstrate the use of SplicePCA, we obtained *Nova2*- knock-out (KO) and wild-type (WT) data from NCBI GEO with the accession number GSE103314 [36]. We performed quality control, trimming, alignment, and alternative splicing analysis as described in Sect.  3.2 RNA-seq analysis.

Subsequently, the cassette exons from the rMATS output file were uploaded to https://cortexa-rna.com/pca and analyzed in the context of developmental [6] and NPC/neuron-specific [9] alternative splicing events. Next, the results from SplicePCA were downloaded and plotted using matplotlib (version 3.9.0) [37].

### Utility and discussion

Alternative splicing is a prevalent regulatory mechanism in the brain that plays an important role during development and in specifying and maintaining neural cell types [6, 7, 9, 22, 28, 36, 38]. However, *Mus musculus* has ~ 22,000 protein-coding genes [39] of which almost all multi-exon genes undergo alternative splicing [40]. Functional implications of these alternative splicing events remain in many cases elusive. Cortexa is an easy-to-use web tool to access alternative splicing events for genes of interest in a developmental and neuronal cell-type-specific context to formulate and investigate research hypotheses.

Additionally, principal component analysis has proven to be a powerful tool to summarize alterations in the alternative splicing landscape [6, 27–29]. Representation of samples in two-dimensional space allows investigating the similarity or divergence of global splicing patterns between different experimental conditions. In our analysis of developmental alternative splicing, we observed a characteristic bell-shaped trajectory across diverse iterations, which aligns with findings reported in the literature [6]. However, the interpretation of principal components can be challenging in terms of associating them with biologically meaningful factors. By design, principal components capture the direction of maximal variance tin the original data [41] which does not necessarily reflect experimental or biological factors. Despite these limitations, PCA remains a valuable exploratory tool, and SplicePCA offers a user-friendly method for investigating alternative splicing in the context of development or different cell types.

Using SplicePCA, researchers can select splice events for specific genes, integrate their data, and interpret results in the context of cortical development and specific neuronal cell types. To showcase the usefulness of this approach, we re-analyzed cortical *Nova2*-KO and WT samples at embryonic day E18.5 [36] and used the SplicePCA tool. NOVA2 belongs to the class of RNA-binding proteins, governing alternative splicing during cortical development and in mature neurons [42]. Specifically, NOVA2 is required to regulate neuronal migration through splicing *Dab1* which is part of the Reelin pathway [2]. By using SplicePCA, we revealed a striking effect of *Nova2*-KO during cortical development. E18.5 knockout samples were located between E14.5 and E16.5 wild-type samples on the inferred developmental splicing trajectory, indicating a less mature splicing pattern (Fig. 2b). Thus, NOVA2 splicing activity contributes significantly to the splicing changes between E14.5 to E18.5, as reported previously [2, 36, 43]. These results support the relevance of SplicePCA which combines available datasets with new datasets. Cortexa thus allows the use of publicly available data without extensive re-analysis, which would otherwise require significant computational resources.

## Conclusions

In summary, Cortexa gives access to high-quality, publicly available transcriptomic data to a broad range of scientists without the need to gain expertise in the computational aspects of gene expression and alternative splicing analysis. For experienced users, SplicePCA offers a powerful tool to summarize alterations in the alternative splicing landscapes upon experimental manipulation and compare results to the normal splicing trajectory during cerebral cortical development or in distinct neuronal cell types. Ultimately, Cortexa offers an easy-to-use extensive platform for gaining novel insights into gene expression and alternative splicing regulatory processes of the mouse brain.

## Data Availability

Publicly available sequencing data were downloaded from NCBI SRA or GEO respectively. Specifically, we used SRP0550086, GSE13329122, and GSE969509. The Nova2-KO data that were used to test SplicePCA and a tutorial are available on GitHub at https://github.com/s-weissbach/cortexa_SplicePCA_example/. All processed datasets used in this study are available on Zenodo at https://zenodo.org/records/13170518.
